# Management of Medication-Related Osteonecrosis of the Jaw in Multiple Myeloma Patients With Pentoxifylline and Tocopherol: Case Reports

**DOI:** 10.1155/crid/2765925

**Published:** 2025-05-07

**Authors:** Adepitan A. Owosho, Katherine A. DeColibus, Layne C. Levy, Osariemen Okhuaihesuyi

**Affiliations:** ^1^Department of Diagnostic Sciences, College of Dentistry, The University of Tennessee Health Sciences Center, Memphis, Tennessee, USA; ^2^Missouri School of Dentistry and Oral Health, A. T. Still University, Kirksville, Missouri, USA

**Keywords:** BRONJ, denosumab, medical management, MRONJ, oncology, zoledronic acid

## Abstract

Medication-related osteonecrosis of the jaw (MRONJ) is a well-known side effect of bone-modifying agents such as antiresorptive medications including pamidronic and zoledronic acids (intravenous bisphosphonates) and denosumab (anti-RANK ligand humanized monoclonal antibody). The major risk factor for the precipitation of MRONJ in a patient taking antiresorptive medication is dentoalveolar trauma such as dental extractions. Management of MRONJ in oncology patients is exceptionally challenging. In this report, two multiple myeloma patients with longstanding advanced-stage MRONJ were successfully managed with combined pentoxifylline–tocopherol treatment pre- and postextraction/sequestrectomy. In conclusion, based on this report and other published reports, it appears that the use of combined pentoxifylline–tocopherol protocol in the management of MRONJ is effective.

## 1. Introduction

The most common cancers in the United States, lung, prostate, and breast cancers, respectively [1], usually metastasize to the bone [2]. The rates are as follows: 65%–90% of prostate cancers, 65%–75% of breast cancers, 17%–64% of lung cancers, and 70%–95% of multiple myelomas metastasize to the bone [2–4]. Patients with metastases to the bone may experience skeletal-related events (SREs), such as uncontrolled pain requiring bone surgery and/or radiotherapy, hypercalcemia, spinal cord injury, and pathological fractures [5]. These SREs necessitate the use of bone-modifying agents such as antiresorptive medications including pamidronic and zoledronic acids (intravenous bisphosphonates) and denosumab (anti-RANK ligand humanized monoclonal antibody). These bone-modifying agents are well known to be associated with medication-related osteonecrosis of the jaw (MRONJ) [6–8]. The major risk factor for the precipitation of MRONJ in a patient taking antiresorptive medication is dentoalveolar trauma such as dental extractions and other forms of dentoalveolar surgeries [9, 10]. The rate of developing MRONJ after a dental extraction amongst oncology patients taking antiresorptive medications ranges from 1.6% to 14.8% [11–13].

Management of MRONJ still remains challenging with no specific therapeutic approach and varying successful outcomes [7]. Pentoxifylline and tocopherol (PENTO) medications were initially used in the management of osteoradionecrosis (ORN), a condition similar to MRONJ but with a different etiology [14, 15]. Given its success in managing ORN, Epstein et al. applied it to the first set of MRONJ patients, and it has since been reported in a few other articles [16–22]. PENTO tends to work via the antioxidant pathway by their antifibrotic synergistic effect in reversing radiation-induced fibrosis as elicited by Delanian and Lefaix [23]. In this report, two multiple myeloma patients with longstanding advanced-stage MRONJ were successfully managed with PENTO pre- and postextraction/sequestrectomy.

## 2. Case 1

A 59-year-old female patient was referred to the Dental Oncology Clinic of the faculty dental practice of the University of Tennessee Health Science Center because of nonhealing extraction sites, recurrent infections, discharge, and pain of the left maxilla of 2 years duration. The patient's medical history was positive for multiple myeloma (ISS: Stage I; cytogenetics: FISH 1q+, 13q del, hyperdiploidy, and IgH deletion), Type II diabetes mellitus, hypertension, hyperlipidemia, and chronic kidney disease. The patient started on 4 mg IV zoledronic acid every month from April 2021 until March 2023 (24 doses). The treatment was discontinued due to oral issues, including nonhealing extraction sites, recurrent infections, discharge, and jaw pain, which arose following the 2022 extractions of carious Teeth #13 and #14 by a dentist in the community. These extractions were performed without interrupting the IV zoledronic acid therapy.

**Figure 1:**
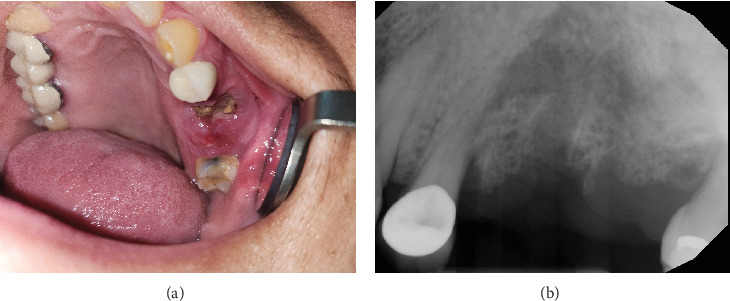
A 59-year-old female with medication-related osteonecrosis of the jaw. (a) The exposed necrotic bone of the left maxillary alveolar ridge measuring 15 mm × 10 mm in size and (b) periapical radiograph showing nonhealing extraction sites with mixed radiopaque and radiolucent areas.

At the time of her presentation to the dental oncology clinic in February 2024, 2 years after the extractions, the patient's complaints were as follows: nonhealing extraction sites, recurrent infections, discharge, and pain of the left maxilla. On extraoral examination, there was left facial fullness with no erythema or facial tenderness. Intraoral examination revealed halitosis, swelling and erythema in the area of missing Teeth #13 and #14, and associated tenderness and purulent discharge upon palpation. The exposed necrotic bone measuring 15 mm × 10 mm was present, and the area of the exposed bone was not loose. The periapical radiograph showed nonhealing extraction sites with mixed radiopaque and radiolucent areas (Figure 1). Considering the patient's medical history, examination, and radiographic findings, a diagnosis of MRONJ Stage 2 was rendered according to the American Association of Oral and Maxillofacial Surgeons' Position Paper on Medication-Related Osteonecrosis of the Jaws-2022 Update.

**Figure 2:**
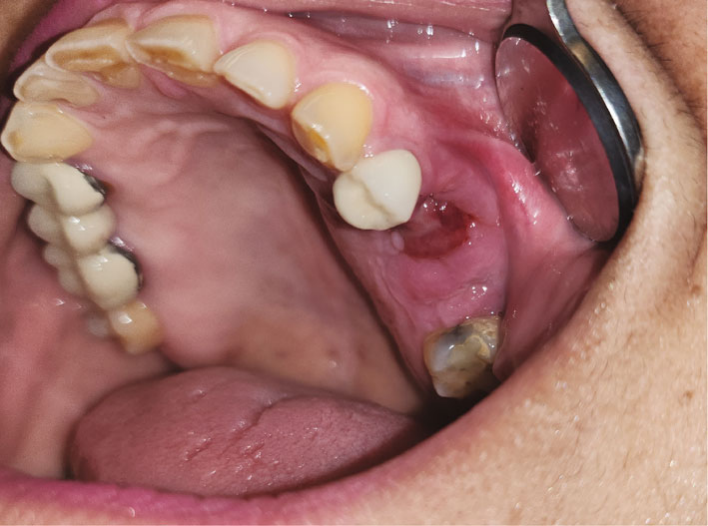
A 59-year-old female with medication-related osteonecrosis of the jaw. Six weeks after using pentoxifylline and tocopherol. Patient reported resolution of symptoms (no pain, discharge, or bone exposure).

**Figure 3:**
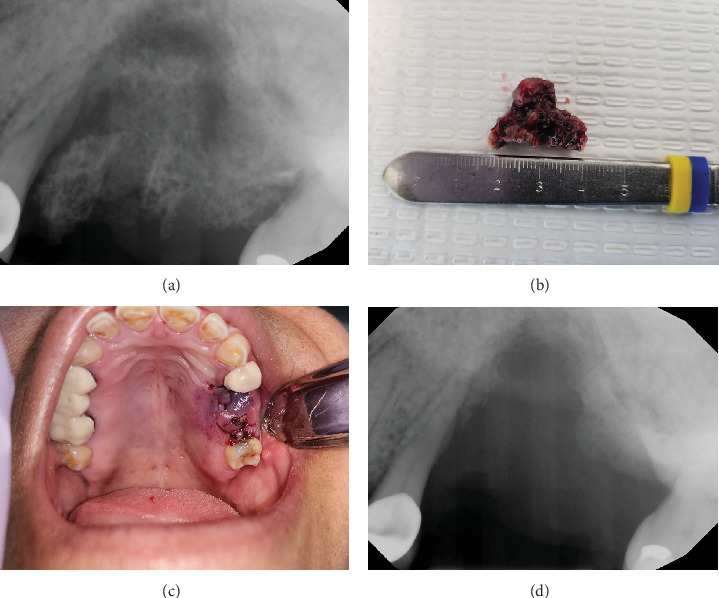
A 59-year-old female with medication-related osteonecrosis of the jaw. (a) A periapical radiograph showing a radiolucent rim separating the sequestrum from the healthy bone. (b,c) A sequestrectomy was performed, and the sequestrum measured 22 mm × 12 mm in size. (d) Postsequestrectomy periapical radiograph.

**Figure 4:**
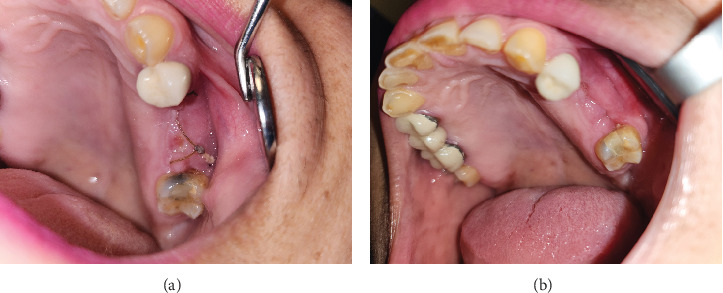
A 59-year-old female with medication-related osteonecrosis of the jaw on pentoxifylline and tocopherol before and after a sequestrectomy. (a) A week after the sequestrectomy procedure showing complete mucosal healing and (b) 6 weeks after the sequestrectomy procedure showing complete mucosal healing.

The area of the exposed necrotic bone was irrigated and debrided with 0.12% chlorhexidine gluconate. The patient was prescribed pentoxifylline 400 mg BID for 4 months, tocopherol 400 IU BID for 4 months, and 0.12% chlorhexidine gluconate rinse six times per day for 2 weeks. The patient presented for follow up every 2–3 weeks, at which time the area was irrigated with 0.12% chlorhexidine gluconate when required. The patient reported resolution of symptoms (no pain, discharge, or bone exposure) (Figure 2, 6 weeks after initial presentation). Twelve weeks after her initial presentation, the patient independently discontinued the PENTO medication, believing her symptoms had resolved and the area healed, without informing her dental oncologist. Subsequently, she developed pain from the area, and she decided to apply peppermint and salt to the area. At her next appointment, the area presented with an exposed necrotic bone. On palpation, the necrotic bone segment was loose, and a periapical radiograph showed a radiolucent rim separating the sequestrum from the healthy bone. The decision was made to perform a sequestrectomy, and the sequestrum measured 22 mm × 12 mm (Figure 3). The patient was instructed to resume the use of PENTO and prescribed an antibiotic for 1 week (875 mg of Augmentin BID). The subsequent follow-up visit showed complete mucosal healing and resolution of symptoms (Figure 4). The patient continued the use of PENTO for 8 more weeks after the removal of the sequestrum and tolerated the medications throughout its use without any adverse effect.

## 3. Case 2

A 57-year-old female patient was referred to the Dental Oncology Clinic at the University of Tennessee Health Science Center due to a nonhealing extraction site, recurrent infections, discharge, and pain in the right mandible for the past year. The patient's medical history was positive for multiple myeloma (ISS: Stage II). The patient started on 120 mg denosumab (Xgeva) once every 4 weeks from March 2023 until March 2024 (12 doses). The treatment was discontinued due to oral issues, including nonhealing extraction sites, recurrent infections, discharge, and jaw pain, which arose following the November 2023 extraction of a carious Tooth #28 by a dentist in the community. This extraction was performed without discontinuing the denosumab therapy.

**Figure 5:**
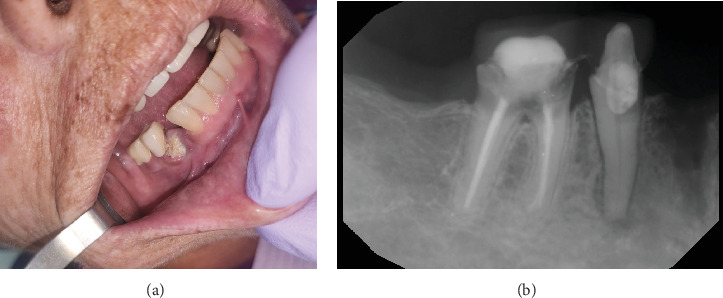
A 57-year-old female with medication-related osteonecrosis of the jaw. (a) The exposed necrotic bone of the right mandibular alveolar ridge measuring 10 mm × 10 mm in size and (b) periapical radiograph showing a nonhealing extraction site of #28 area of a year duration, secondary decay and periapical radiolucency of Tooth #29, and secondary decay with furcation involvement of an endodontically treated Tooth #30.

At the time of her presentation to the dental oncology clinic in July 2024, a year after the extraction, the patient's complaints were nonhealing extraction site, recurrent infections, discharge, and pain of the right mandible. Intraoral examination revealed a 10 mm × 10 mm area of the exposed necrotic bone that was tender to palpation but not loose. The periapical radiograph showed a nonhealing extraction site (Figure 5). Considering the patient's medical history, examination, and radiographic findings, a diagnosis of MRONJ Stage 2 was rendered according to the American Association of Oral and Maxillofacial Surgeons' Position Paper on Medication-Related Osteonecrosis of the Jaws-2022 Update. Additionally, Tooth #29 was very painful with secondary decay and periapical radiolucency, and Tooth #30 had secondary decay with furcation involvement.

**Figure 6:**
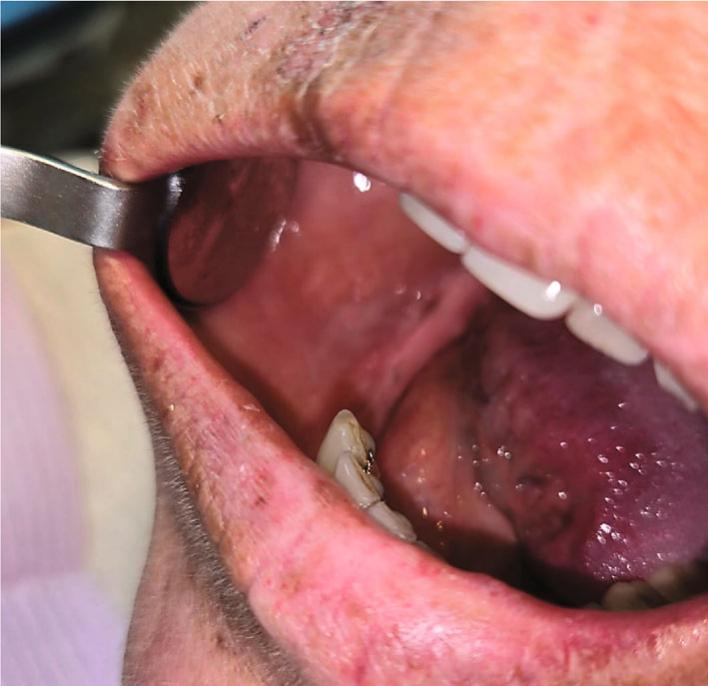
A 57-year-old female with medication-related osteonecrosis of the jaw on pentoxifylline and tocopherol. Twelve weeks after extractions, alveolectomy and sequestrectomy procedures show complete mucosal healing.

After discussing with the patient, it was decided to address Teeth #29 and #30, possibly through extractions. The necrotic bone area was irrigated and debrided with 0.12% chlorhexidine gluconate. She was prescribed pentoxifylline 400 mg BID for 4 months, tocopherol 400 IU BID for 4 months, and 0.12% chlorhexidine gluconate rinse six times a day for 3 weeks. After 3 weeks, Teeth #29 and #30 were extracted and alveolectomy and sequestrectomy of the necrotic bone in the area of #28 were performed. The gingival mucosal tissue was sutured closed. She was instructed to continue the PENTO medications and was prescribed an antibiotic (875 mg of Augmentin BID) for 1 week. Follow-up visits were scheduled every 2 weeks. At the 6-week follow-up, she showed complete mucosal healing and resolution of symptoms (Figure 6). She completed the 4-month PENTO regimen, but during the course of use, she was experiencing nausea, so the dose regimen was adjusted to 800 mg of pentoxifylline and 800 IU of tocopherol once at night, which she tolerated well.

## 4. Discussion

The use of PENTO in the management of MRONJ was first reported by Epstein et al., and since then, only five other reports/series have been published [16–21]. Recently, a prospective randomized clinical trial was published of 202 MRONJ osteoporotic patients by Colapinto et al. [22]. The study by Colapinto et al. divided the 202 MRONJ patients into a test group (108 patients) and a control group (94 patients). All test patients prescribed PENTO pre- and postsequestrectomy healed completely except for one patient who relapsed within 6 months. However, 95% of all control patients (not prescribed PENTO pre- and postsequestrectomy) either did not heal, relapsed within 2 months, or developed microbial complications. These control patients were then placed on PENTO, and every one of them healed completely [22]. A laboratory study on the effect of PENTO in preventing and managing MRONJ in rats showed that the PENTO protocol was significantly effective in reducing the incidence of MRONJ and the presence of bone sequestrum and increasing alveolar bone repair and alveolar blood flow in rats that received 4 weeks of PENTO after the discontinuation of antiresorptive medication [24]. In this report, we described the use of PENTO in the management of refractory cases of MRONJ which were present for 2 years and 1 year, respectively, before referral to the first author's dental oncology clinic. A few weeks after beginning the PENTO medications, the patients reported improvement of symptoms (no pain, discharge, or bone exposure). The choice of dosages and duration for pentoxifylline (400 mg BID) and tocopherol (400 IU BID) is informed by the first author's personal experience and other reported literature on using PENTO medications prophylactically to prevent ORN of the jaw following invasive dental procedures in postradiated oral and oropharyngeal cancer patients [25].

While there is no consensus on the management of MRONJ, different therapeutic approaches (antimicrobials [antiseptics and antibiotics], teriparatide, hyperbaric oxygen, PENTO, photobiomodulation, photodynamic therapy, surgeries aided with fluorescence light or platelet-rich plasma protein, and various surgical options) are utilized in the management of MRONJ with varying degrees of successful outcomes [21, 26–29]. PENTO is a safe, readily available, easily prescribed, better tolerated, and a less expensive management option. The study by Magalhaes et al. showed the prophylactic benefit of PENTO in the prevention of MRONJ after dental extraction in cancer patients on antiresorptive medications [30].

The PENTO protocol should be a revolutionary development in clinical oncology practice. The emphasis has been on the prevention of MRONJ which entails premedication dental evaluation (patient education, comprehensive dental evaluation, and completion of recommended dental treatment), which is highly encouraged [31–34]. However, in some oncology/dental practices, patients are emboldened to extract all their teeth with the slightest pathoses, before beginning antiresorptive therapy. Many dental care providers are not comfortable carrying out invasive dental procedures such as root planning and extractions in oncology patients who have been on antiresorptive medications. As such, many of these oncology patients must endure complications such as dentoalveolar infections, cellulitis, and jawbone osteomyelitis. The major setback of the use of pentoxifylline is the adverse effect of the medication on the gastrointestinal system causing nausea, vomiting, irritation, and epigastralgia especially if used for a long duration. We suggest conducting extensive prospective studies to further confirm these results.

## Data Availability

Data sharing is not applicable to this article as no datasets were generated or analysed during the current study.
